# Structure of *Mycobacterium thermoresistibile* GlgE defines novel conformational states that contribute to the catalytic mechanism

**DOI:** 10.1038/srep17144

**Published:** 2015-11-30

**Authors:** Vitor Mendes, Michal Blaszczyk, Ana Maranha, Nuno Empadinhas, Tom L. Blundell

**Affiliations:** 1 Department of Biochemistry, University of Cambridge, Cambridge CB2 1GA, UK; 2 Molecular Mycobacteriology Group, CNC-Center for Neuroscience and Cell Biology, University of Coimbra, 3004-517 Coimbra, Portugal

## Abstract

GlgE, an enzyme of the pathway that converts trehalose to α-glucans, is essential for *Mycobacterium tuberculosis*. Inhibition of GlgE, which transfers maltose from a maltose-1-phosphate donor to α-glucan/maltooligosaccharide chain acceptor, leads to a toxic accumulation of maltose-1-phosphate that culminates in cellular death. Here we describe the first high-resolution mycobacterial GlgE structure from *Mycobacterium thermoresistibile* at 1.96 Å. We show that the structure resembles that of *M. tuberculosis* and *Streptomyces coelicolor* GlgEs, reported before, with each protomer in the homodimer comprising five domains. However, in *M. thermoresistibile* GlgE we observe several conformational states of the S domain and provide evidence that its high flexibility is important for enzyme activity. The structures here reported shed further light on the interactions between the N-terminal domains and the catalytic domains of opposing chains and how they contribute to the catalytic reaction. Importantly this work identifies a useful surrogate system to aid the development of GlgE inhibitors against opportunistic and pathogenic mycobacteria.

Mycobacteria comprise a diverse group of organisms, many of which are known human pathogens. The most notorious member of this genus is undoubtedly *Mycobacterium tuberculosis*, the major causative agent of tuberculosis (TB), a disease that remains a leading cause of mortality worldwide, claiming an estimated 1.5 million lives in 2013^1^. Furthermore, infections by opportunistic non-tuberculous mycobacteria (NTM) are increasing worldwide due to their colonization of man-made environments, such as water distribution systems, and their intrinsic detergent and drug resistance that allows them to flourish in hospital environments^2^.

Despite available treatments the rapid emergence of multi-drug and extensively-drug-resistant strains, together with a deadly synergy with HIV, has severely limited our capacity to control and eradicate TB. The combination of the recent increase of NTM infections with the appearance of totally drug-resistant TB strains^3^ makes the finding of new tools to develop new drugs against these organisms a priority.

GlgE is a maltosyl transferase that transfers maltose from a maltose-1-phosphate (maltose-1P) donor to α-(1–4) glucan chains^4^,^5^. This enzyme is part of a pathway involving three other enzymes, trehalose synthase (TreS), maltokinase (Pep2/Mak) and glycogen branching enzyme (GlgB), which link trehalose recycling to glycogen and other branched α-glucans synthesis^4^,^5^,^6^. GlgE is essential for *M. tuberculosis* and its depletion leads to pleiotropic stress effects and eventually cell death^4^. Interestingly, cell death is associated with substrate (maltose-1P) hyper-accumulation, and not with the absence of product, since there are at least two other pathways in *M. tuberculosis* that lead to glycogen and other α-glucan synthesis^4^,^7^. There appears to be complex crosstalk between the GlgE pathway, Rv3032 of the methyl glucose lipopolysaccharide (MGLP) pathway, and glycogen synthase/glucose-1-phosphate adenylyltransferase (GlgA/GlgC) classical glycogen pathway^4^,^8^,^9^, with a synthetic lethal interaction being reported for TreS and Rv3032^4^. This crosstalk seems to be at least partially regulated by phosphorylation, with serine/threonine protein kinase PknB playing a significant role in regulating GlgE activity^10^. Additionally, the TreS-Pep2-GlgE-GlgB pathway is distributed almost exclusively in bacteria, with rare exceptions in archaea and with no known GlgE orthologues in eukaryotes^7^. The combined essentiality of GlgE with the lack of orthologues in humans makes it a very attractive target for TB drug discovery.

GlgE belongs to the glycosyl hydrolase GH13_3 family^11^. GH13 enzymes exhibit a conserved three-domain core usually designated by A, B and C^12^. Domain A is a (β/α)_8_ barrel, while a small domain of variable size (domain B) is inserted between β3 and α3 of domain A^13^. Domain C is a β-sandwich domain located at the C-terminus^13^. The active site of GH13 enzymes is found in a cleft between domains A and B^14^. The structure of *Streptomyces coelicolor*, GlgE isoform I, a 5-domain enzyme forming a dimer, exhibits the typical GH13 core domains with two extra domains at the N-terminus: an α-helix bundle and a second β-sandwich domain^15^.

GlgE is proposed to catalyse the transfer of maltosyl units to α-glucan chains through a double-displacement reaction in which an aspartate residue acts as a nucleophile forming an β-maltosyl-enzyme intermediate, while a glutamate residue is thought to protonate the phosphate and deprotonate the acceptor, acting like an acid/base catalytic residue^16^. Furthermore, the first GlgE inhibitors have been reported recently^17^,^18^ and the first low resolution *M. tuberculosis* GlgE structures have just become available^19^.

We here report the first high-resolution X-ray analyses of a mycobacterial GlgE, defining a maltose-GlgE complex of *Mycobacterium thermoresistibile* GlgE, as well as the apo form and a new conformational state obtained by co-crystallizing GlgE with maltose-1P. We show that *M. thermoresistibile* GlgE structure resembles that of *S. colelicolor* and *M. tuberculosis* GlgEs with each protomer in the dimer comprising five domains, but in *M. thermoresistibile* GlgE we observe several conformational states of the S domain that point towards considerable flexibility and shed further light on its role in the catalytic mechanism.

## Results

### Kinetic properties of M. thermoresistible GlgE

We first determined kinetic parameters in order to compare enzyme properties with *M. tuberculosis* and with *S. coelicolor* GlgE, the only orthologous structures available to date. As with *M. tuberculosis* and *S. coelicolor* GlgE^4^,^15^, *M. thermoresistibile* GlgE uses maltose-1P exclusively as a maltosyl donor. *M. thermoresistible* GlgE *K*_M_ and *k*_cat_ values are comparable to those of orthologues from *M. tuberculosis and S. coelicolor* GlgE (Table 1), with *K*_M_ values of 0.29 ± 0.04 mM for maltose-1P in the presence of 1 mM maltohexaose and of 7.09 ± 0.94 mM for maltoheaxose in the presence of 5 mM maltose-1P (Supplementary Fig. S1). Both calculated constants are in line with previous observations in *M. smegmatis* when glycogen was used as a maltosyl acceptor^5^.

### *M. thermoresistibile* GlgE overall structure

The three different structures obtained in this study with resolution limits ranging from ~2.0 to ~3.3 Å were GlgE apo-form (5CJ5), GlgE-maltose complex (5CGM) and a second GlgE-maltose complex (5CIM) obtained by co-crystallizing with GlgE with maltose-1P. Of the three different tags (C-terminal non-cleavable 6xHis Tag, N-terminal non-cleavable 6xHis Tag and N-terminal cleavable SUMO tag) used, the construct with an N-terminal cleavable SUMO tag produced the best diffracting crystals and was used to obtain crystals in all conditions. However, we could only obtain high-resolution crystals (~2.0 Å) when GlgE was co-crystalized with maltose, maltohexaose or both. Apo-form crystals and GlgE-maltose-1P co-crystallizations always produced crystals that diffracted to lower resolutions (<3 Å) despite all optimization efforts. The maltohexaose and maltose co-crystallizations produced indistinguishable structures in which only maltose could be observed at the active site. This was not unexpected since others have reported that GlgE slowly degrades maltooligosaccharides to maltose in the time scale required for crystallization^15^. Therefore we present only the highest resolution structure obtained in these conditions.

The structure of *M. thermoresistible* GlgE was solved by molecular replacement at 1.96 Å (see methods) and refined with a final R-factor of 0.175 and *R*_free_ of 0.203 (Table 2). This model proved useful for solving the structures of apo form and maltose-1P co-crystallization condition by molecular replacement at 3.13 and 3.32 Å respectively. Both structures have been refined to acceptable statistics (Table 2).

**Figure 1:**
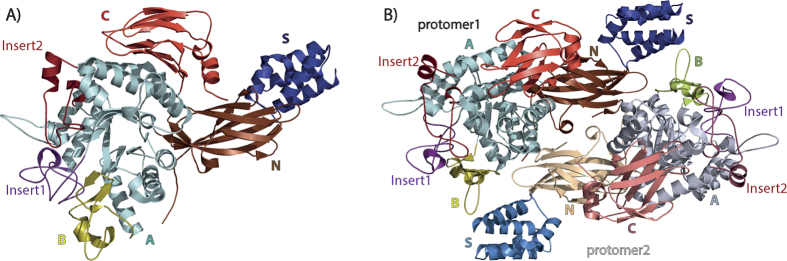
(**A**) Cartoon representation of the overall structure of M. *thermoresistibile* GlgE. Loops have been simplified for clarity. Domains N consists of residues 1–122 and 205–220, domain S 123–204, domain A 221–273, 319–340, 387–530 and 569–590, domain B 341–386, domain C 591–696, insert 1 274–318 and insert 2 531–568. (**B**) Cartoon representation of *M. thermoresistibile* GlgE dimer. Figure was prepared with Pymol (http://www.pymol.org).

**Figure 2:**
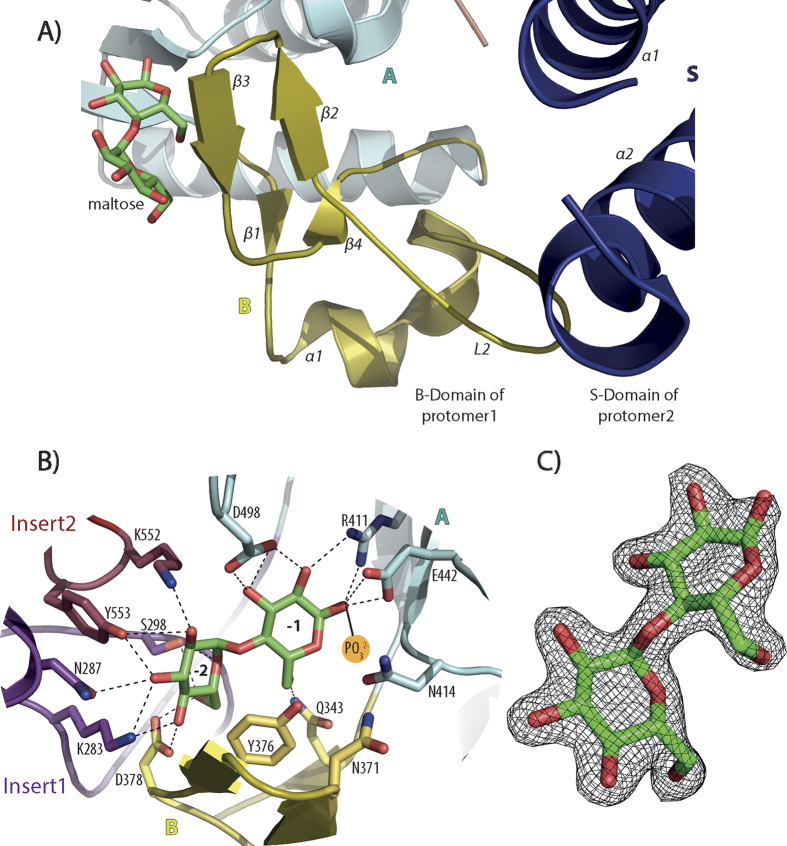
(**A**) Close view of domain B and S of opposing protomers of M. *thermoresistibile* GlgE with maltose bound. (**B**) View of the active site of *M. thermoresistibile* GlgE with maltose bound. Individual domains are represented in different colours. Dashed black lines represent hydrogen bonds. Subsites −1 and −2 are highlighted (**C**) Difference electron density map “omit map” of maltose. This map was generated using the phases from the final model.

*M. thermoresistibile* GlgE forms a dimer in all obtained structures and in solution, a feature reported previously for GlgE in other studied organisms^4^,^5^,^15^. Each of the protomers of *M. thermoresistibile* GlgE contains 5 domains and 2 inserts (Fig. 1) that have been described extensively in the first reported GlgE structure^15^ and therefore we will focus on the particular characteristics of *M. thermoresistibile* GlgE. The N-terminal, domain N is a β-sandwich domain, responsible for the majority of dimerization contacts and interacting directly with the catalytic domain of the adjacent protomer. This domain contains a very long loop of 36 residues, connecting β-strands 4 and 5 of this domain, part of which (residues 71–86) has no observable density and therefore was not modelled. This loop is highly variable both in length and amino acid composition even among closely related mycobacterial species (Supplementary Fig. S2). Domains S and B also contribute to the dimer interface but to a lesser extent (Fig. 1 and Fig. 2A). Domain A and B together with inserts 1 and 2 form the catalytic unit. The C-terminal domain (domain C), a second β-sandwich domain, is not directly involved in catalytic activity but sits on top of the catalytic domain A, as reported before in *S. coelicolor* GlgE^15^. A C-terminal short α-helix in the *M. thermoresistibile* domain C, which contacts domain N, is found in all other mycobacterial GlgEs but is not found in that of *S. coelicolor* (Supplementary Fig. S2). Domain C together with domain A were recently shown to to be involved in the binding of α-glucan chains^19^. Although the identified binding patch in the surface of the protein is not 100% conserved there is a high degree of conservation among mycobacterial species (Supplementary Fig. S2).

**Figure 3:**
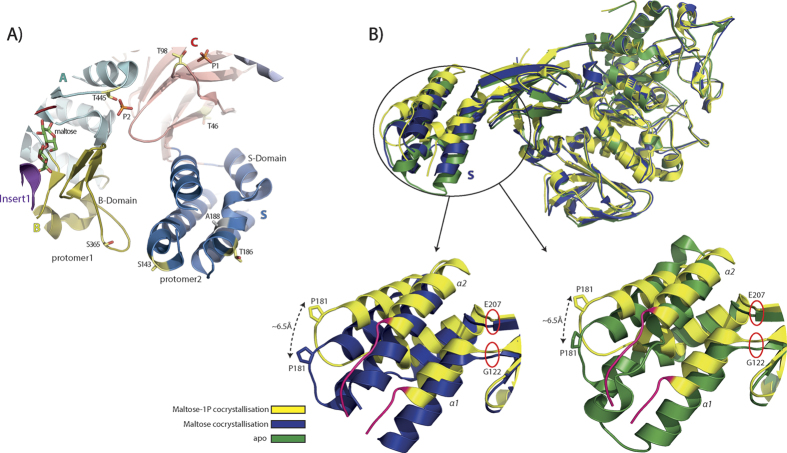
(**A**) View of M. tuberculosis GlgE phosphorylation sites mapped in M. *thermoresistibile* GlgE structure (5GCM). T46 and T186 are conserved in *M. tuberculosis* and *M. thermoresistibile*. S143 and S365 are mutated to threonine in *M. tuberculosis*. A188 and M80 are mutated to serine in *M. tuberculosis*. The side chain of S143 and the loop where M80 is located were not modeled since electron density is poor in those regions. The putative phosphorylation site T98 and T445 are also highlighted. (**B**) Superposition of structures of maltose co-crystallization (5GCM), maltose-1P co-crystallization (5ICM) and apo form (5CJ5). The represented protomer of maltose-1P structure has maltose bound. Regions that lose helical conformation in the maltose-1P co-crystallization structure are highlighted in red.

In the maltose co-crystallization structure (5CGM) several ethylene glycol molecules and polyethylene glycol chains of different sizes are visible due to PEG300 being present in the crystallization condition at high concentration (30%). Two phosphate groups can also be seen bound to each of the chains sitting in a groove between domain N and the terminal α-helix of C. Two other phosphate groups sit in a positively charged patch formed by domains A and N of the two opposing protomers, near the active site. Interestingly all the phosphate groups are close to threonines, T98 for the first duo and T445 for the second (Fig. 3).

The apo form structure (5CJ5) is similar to the maltose-containing structure, with the most significant differences occurring in domains S and C where shifts are observed of ~4.3 and ~7.5 Å respectively (Supplementary Fig. S3). However, apo-form crystals diffracted at a much lower resolution than the maltose co-crystallization condition. Electron density cannot be distinctively seen for residues 598–603, 621–635 and 663–669 of the domain C of chain A in this structure due to direct neighbouring with symmetry element. The same residues are clearly visible in Chain B where the β-sandwich fold of domain C is seen intact.

### Catalytic site

The active site of GlgE sits at the TIM-barrel domain, but domain B together with inserts 1 and 2 are also part of the active site^15^ (Fig. 2B). From the maltose-bound structure, which is the highest resolution structure reported in this work, we can see that the donor substrate forms hydrogen bonds with the side chains of the highly conserved K283, N287 and S298 of the insert 1. Interestingly S298 is not conserved in *S. coelicolor* GlgE, where it is replaced by a valine, but conservation of serine occurs in all mycobacteria and in almost all other GlgEs. This serine forms hydrogen bonds with the C6 hydroxyl group (~2.8 Å) and the ring oxygen (~3.1 Å) of the non-reducing glucose. K283 forms hydrogen bonds with the C3 and C4 hydroxyl groups and N287 with C2 hydroxyl group, all from the non-reducing glucose. The donor substrate further interacts with the side chains of the absolutely conserved Q343 and D378 of domain B, R411, D413, E442 and D498 of domain A and K552 and Y553 of insert 2 (Fig. 2B). Three other residues (N371, Y376 and N414) have been shown previously to form hydrogen bonds with the phosphate group^16^. Although in this work we did not obtain a structure with intact maltose-1P (see below), these three residues are present in all mycobacterial GlgEs and conserved in all GlgE sequences.

### Maltose-1P-GlgE co-crystallization

**Figure 4:**
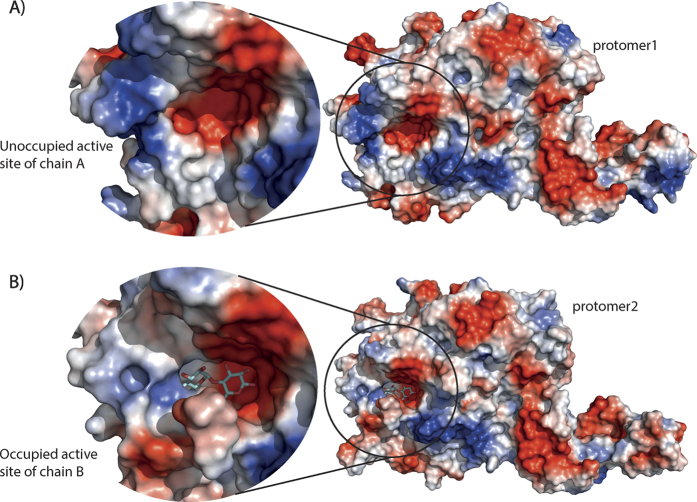
Comparison of the active sites of chain A and B of the maltose-1P co-crystallization condition. Maltose is only present in chain B. The antiparallel β-strand lid and loop 2 of domain B are only visible in chain B.

In an attempt to obtain a maltosyl-GlgE intermediate state, GlgE was co-crystalized with maltose-1P. We could not observe the maltosyl-GlgE intermediate but instead we found maltose bound to only one of the active sites of the dimer (Fig. 4). The fact that maltose was present instead of maltose-1P is most likely due to GlgE slowly degrading maltose-1P to maltose, since maltose-1P was free of maltose contamination and we confirmed its stability at different temperatures for long periods of time (Supplementary Fig. S4). Moreover others have also reported slow degradation of maltose-1P by *S. coelicolor* GlgE during the crystallization time span^15^. Significant conformational changes were however visible in the structure especially in the S domain of the protomer with bound maltose (Fig. 3B and Supplementary Fig. S5A). The S domain of this protomer exhibits conformational changes with several residues of α1 (V132–L136) and α2 (S146–150) losing their helical conformation (Fig. 3B). These residues are those closer to loop 2 of domain B of the “apo” protomer and the observed changes can indicate differences in the contacts between these two domains (Supplementary Fig. S5A). Moreover, all four helices comprising the S domain of the maltose-bound protomer were found to be shifted with a maximum distance of ~6.5 Å when compared to both the maltose co-crystallization and the apo structure. This brings domain S of the maltose containing protomer closer to loop 2 of domain B of the “apo” protomer (Fig. 3). These observations imply a mechanism where domain S of one protomer interacts with loop 2 of domain B of the opposing protomer to move the antiparallel β-strand lid that covers the active site into an open or closed conformation, allowing the substrate/product to enter/leave the active site. Unfortunately, the majority of domain B, including loop 2 and the antiparallel β-strand lid, is not seen clearly in the maltose-1P co-crystallization structure “apo-protomer” and thus it could not be modeled (Fig. 4 and Supplementary Fig. S5A).

## Discussion

The overall structure of the *M. thermoresistibile* GlgE is very similar to the previously reported structures of *M. tuberculosis* and *S. colelicolor* GlgEs^15^,^19^ with each of the protomers in the dimer comprising five domains (Supplementary Fig. S5B). The major structural differences between the mycobacterial and the *S. coelicolor* structures occur in the relative positions of the S domain helices and in the presence of an extra C-terminal alpha helix in mycobacterial GlgEs. Although in the previously reported *S. coelicolor* and *M. tuberculosis* GlgE structures^15^,^16^,^19^,^20^ no significant differences are observed between ligand-bound structures and apo form, in our *M. thermoresistibile* GlgE structures we observe several conformational states indicating a large flexibility of the S domain that hinges at residues Gly122 and Glu207 (Fig. 3B). We could not obtain a maltosyl-GlgE intermediate when GlgE was co-crystallized with maltose-1P, due to the slow but continuous degradation of maltose-1P by GlgE^15^, even though our initial GlgE-maltose-1P ratio in the crystallization drop was ~1:60. To address this issue others have produced mutated *S. coelicolor* GlgE and used 2-deoxy-2-fluoro-α-maltosyl fluoride and have been successful in obtaining a structure of this intermediate state and also a maltose-1P-GlgE complex^16^. Nevertheless our co-crystallization with maltose-1P allowed us to obtain an intermediate state where only one of the protomers of the dimer contains maltose. The significant differences found between the two S domains (apo and maltose bound) (Fig. 3B) point to a mechanism where the S domain interacts with the domain B of the opposite protomer to open/close the antiparallel β-strand lid covering the active site. Unfortunately the majority of domain B, including loop 2 and some regions of insert one of the “apo” protomer in the maltose-1P co-crystallization structure (5CIM), could not be modelled due to lack of electron density (Supplementary Fig. S5A). This could be explained by an increased flexibility of the catalytic domain when in an open/relaxed state before binding of maltose-1P. These differences cannot be dismissed as crystallographic artefacts since both maltose and maltose-1P-co-crystallization structures were obtained in the same conditions and crystals belong to the same space group with same packing and similar unit cell dimensions (Table 2).

PknB, a serine/threonine protein kinase, was reported to play a fundamental role in regulating GlgE by substantially decreasing GlgE activity by phosphorylation of specific residues^10^. The phosphorylation sites have been mapped for *M. tuberculosis* GlgE, however not all of them are conserved in *M. thermoresistibile* (Supplementary Fig. S2). Amongst those not conserved two threonines are mutated to serine in *M. thermoresistibile* and it is likely that they will be phosphorylated by PknB. Nevertheless, the fact that two of them are located in domain S and one in loop 2 of domain B is consistent with an interaction of these two domains that is important for the catalytic activity of GlgE. Although it has been found that other *Actinomycetes* besides *M. tuberculosis* also phosphorylate GlgE and thus might regulate GlgE activity by phosphorylation^10^, it is interesting to note that many of the phosphorylation sites are not fully conserved. This raises questions about the significance of the regulation of GlgE activity by phosphorylation in other species and whether there are other/different phosphorylation sites in those species. In the high resolution structure we are reporting (5GCM) we could observe four phosphate groups that are close to threonines. One of the threonines, T445 of domain A, sits close to the active site and within what could be the binding region of the acceptor substrate (Fig. 3A). This threonine is highly conserved in all mycobacteria species and even in closely related organisms (Supplementary Fig. S2). It is tempting to speculate that this threonine may also be subject to phosphorylation. Although phosphorylation has not been reported for this particular threonine by Leiba and colleagues, they have restricted their work to PknA to F^10^. Several other Pkn kinases are present in mycobacteria that could potentially phosphorylate GlgE.

Domain C is found in many other members of the GH13 family. It has been proposed to help stabilize the catalytic domain and it could also be involved in substrate binding in some cases^21^. Recently it was shown to be involved in α-glucan chain binding in GlgE^19^. The extra α-helix present in mycobacteria forms direct contacts with the first β-sandwich domain (domain N) and creates a small grove where we observe phosphate binding. This phosphate again interacts with another threonine, T98 in this case. This threonine belongs to the N domain but unlike T445 it is not conserved even within *Mycobacterium* genus. Nevertheless, other mycobacteria and closely related species have a threonine in the terminal α-helix of domain C that is surface exposed and could still form interactions with this phosphate, potentially keeping it in the same area if these threonines would be phosphorylated by a yet unidentified kinase. These contacts and potential phosphorylations sites may also have some indirect influence on the catalytic activity mediated by the two β-sandwich domains. However we do not see major differences in this terminal α-helix between our three structures, nor do we see differences in the domain N that could give some indication of the structural effect of these putative phosphorylation site.

The global interactions with maltose in both donor subsites −2 and −1 are identical for mycobacterial and *S. coelicolor* GlgE with the exception being only the presence of a serine in mycobacteria that is replaced by a valine in *S. coelicolor*. This serine forms hydrogen bonds with the C6 hydroxyl group of the non-reducing hexose ring and further contributes to the highly hydrophilic nature of the active site. To address this difference other groups have mutated this valine to serine in *S. coelicolor* active site to make it more similar to mycobacteria and have successfully used it has a surrogate system^16^,^19^,^20^.

Recently it was shown that several loops of domains A and C form a binding patch for linear α-glucan chains^19^. This patch is not totally conserved even when comparing GlgE sequences within mycobacteria (Supplementary Fig. S2). Nevertheless it was suggested that hydrogen bonds between the linear α-glucan chain and the backbone atoms are more important than side chain interactions^19^. This surface patch sits at 26 Å from the catalytic site and therefore it is not immediately involved in the catalytic reaction. It should however play an important role in orienting an α-glucan chain towards the catalytic site. Some questions still remain unanswered. The specific orientation and binding region as well as what defines specificity of the acceptor substrates are yet to be clarified and although we could see leftover density in maltohexaose co-crystallization structures only a few atoms of a third glucose ring could be modeled (data not shown).

GlgE confirmed essentiality in *M. tuberculosis* and lack of human orthologues makes it an attractive target for drug discovery. The structure we report here will allow more accurate models of this protein to be built for other mycobacteria particularly as no other high resolution mycobacterial structures are available to date. The observations that kinetic constants are intermediate between those found for *M. tuberculosis* and *S. coelicolor* GlgEs and 100% sequence identity at the active site when compared with other mycobacterial GlgEs indicate that it can be used as a good surrogate system for target-based drug discovery in these organisms.

## Methods

### Bacterial strains and cloning

The *glgE* gene was amplified from chromosomal DNA of *M. thermoresistibile* (DSM 44167) obtained from the Deutsche Sammlung von Mikroorganismen und Zellkulturen GmbH (DSMZ, Braunschweig, Germany), with primers based on the sequence retrieved from NCBI database. A BamHI restriction site was added to the forward primer 5′-TAA**GGATCC**GTGGCCGGTCGGATCGTGATC-3′ and a HindIII restriction site to the reverse primer 5′-ATT**AAGCTT**TCACTCCCTGCGCAGCAGTTGC-3′. After amplification, *glgE* was cloned in a pET28a vector (Novagen), modified with an N-terminal 6xHis-SUMO tag, and transformed into *E. coli* BL21(DE3) strain (Invitrogen). The resulting recombinant plasmid was verified by DNA sequencing.

### Recombinant overexpression and protein purification

Transformed *E. coli* BL21(DE3) cells were grown to mid-exponential growth phase (OD_610_ = 0.8) in LB media, containing 30 mg L^−1^ kanamycin at 37 °C. Isopropyl β–D-1-thiogalactopyranoside (IPTG) was then added at a final concentration of 0.5 mM to induce gene expression and the temperature was lowered to 18 °C. Cells were harvested 18 h–20 h later by centrifugation and re-suspended in 50 mM bis-tris propane (BTP) pH 7.2, 0.5 M NaCl and 20 mM imidazole with protease inhibitor tablets (Roche), DNAseI and 5 mM MgCl_2_. Cells were lysed with an emulsiflex (Avestin) and cell lysate was centrifuged at 27000 *g* for 30 mins to remove cell debris.

Recombinant *M. thermoresistibile* GlgE was purified with a HiTrap IMAC Sepharose FF column (GE-Healthcare), equilibrated with 50 mM BTP pH 7.2, 0.5 M NaCl and 20 mM imidazole. Elution was performed in the same buffer with 500 mM imidazole that was subsequently removed with a desalting column followed by SUMO tag overnight cleavage at 4 °C, by adding Ulp1 Protease at 1:100 ratio in 20 mM BTP pH 7.2, 0.5 M NaCl. SUMO tag, Ulp1 protease and uncleaved SUMO-GlgE were removed with a HiTrap IMAC Sepharose FF column (GE-Healthcare), equilibrated with 20 mM BTP pH 7.2, 0.5 M NaCl, 20 mM imidazole. Flow through containing GlgE was collected, concentrated and loaded on a Superdex 200 column equilibrated with 20 mM BTP pH 7.2 and 200 mM NaCl. Fraction purity was determined by SDS-page and purest fractions were pooled, concentrated to ~14.5 mg.ml^−1^ in 20 mM BTP pH 7.2 100 mM NaCl, flash frozen in liquid nitrogen and stored at −80 °C.

### Characterization of recombinant M. thermoresistibile GlgE

Reaction mixtures containing pure recombinant GlgE (0.05–0.5 μM), maltoheaxaose, maltose-1P and 100 mM NaCl in 100 mM BTP buffer pH 7, were incubated at 37 °C and stopped at different time points. Activity was determined by the release of free phosphate, with a quantitative end point assay, using the Ames method^22^. Kinetic parameters were calculated by measuring rates of reaction using a range of substrate concentrations (0.25–10 mM for maltose-1P and 0.5–100 mM for maltoheaxaose). Pure maltose-1P was obtained from ExtremoChem. Data were fitted to the Michaelis-Menten equation using a least squares fitting in Prism 5 (GraphPad Software). All experiments were performed in triplicate.

### Crystallization and X-Ray data Collection

GlgE crystallization screens and optimization were performed using the sitting-drop vapour diffusion method at 291 K with a protein concentration of ~7 mg/ml. Initial crystallization conditions for apo form were obtained from a PACT premier crystallization screen (Molecular Dimensions) solution H12, using an Art Robbins Phoenix crystallization robot by mixing 0.3 μl of protein solution and 0.3 μl well solution. GlgE-ligand complex crystals were obtained in JCSG-plus crystallization screen (Molecular Dimentions) solution C6, using the same protein and well volumes as above but incubating GlgE solution with 5 mM maltose for 30 mins prior to setup crystallization. The optimized conditions consisted of PEG3350 20% (v/v), 0.2 M sodium malonate and 0.1 M BICINE buffer pH 9.5 (apo form) and PEG 300 35% (v/v) and 0.1 M sodium phosphate/citrate buffer pH 4.0 (GlgE-ligand complexes). Other ligands (maltohexaose and maltose-1P) were co-crystallized under the same conditions using ligand concentrations of 5 mM. Crystals were flash frozen in liquid nitrogen and stored for data collection at stations I04, I04–1 and I24 at Diamond Light Source (Oxford, UK). Data collection and refinement statistics are summarized in (Table 2).

### Structure determination and refinement

Diffraction data were processed using MOSFLM^23^ and Aimless^24^ from the CCP4 suite^25^ or autoPROC from Global Phasing Limited^26^. GlgE-ligand complexes crystallized in P2_1_2_1_2_1_ spacegroup, while apo crystals belonged to P6 spacegroup, both with one dimer per asymmetric unit. Analysis with Pointless^27^ suggested that apo form crystallized in P6_3_22 spacegroup, however L-test suggested twinning. XTRIAGE from PHENIX software package^28^ indicated that the crystals were merohedraly twinned with operator h,-h-k,-l and therefore belonged to a lower symmetry spacegroup. Initial phases were determined with PHASER^29^ from PHENIX software package^28^ using the structure of *S. coelicolor* GlgE (PDB entry 3ZSS)^15^ as a search model. Model building was done with Coot^30^ and refinement was performed in PHENIX^28^. Structure validation was performed using Coot and PHENIX tools^28^,^30^. Refined coordinates and structure factors have been deposited in PDB with the accession numbers 5CJ5 (apo form), 5CGM (GlgE-maltose co-crystallization) and 5CIM (GlgE-maltose-1P co-crystallization).

## Additional Information

**How to cite this article**: Mendes, V. *et al.* Structure of *Mycobacterium thermoresistibile* GlgE defines novel conformational states that contribute to the catalytic mechanism. *Sci. Rep.* **5**, 17144; doi: 10.1038/srep17144 (2015).

## Supplementary Material

Supplementary Information

## Figures and Tables

**Table 1:** Comparison of kinetic parameters between *M. thermoresistibile*, *M. tuberculosis* and *S. Coelicolor* GlgEs.

Enzyme	Substrate	*K*_m_(mM)	*k*_cat_ (s^−1^)	*k*_cat_/*K*_m_(M^−1^s^−1^)
*M. thermoresistibile*	Maltose-1P^a^	0.29 ± 0.04	4.06 ± 0.11	14000 ± 2800
	Maltoheaxaose^a^	7.09 ± 0.94	49.91 ± 1.73	7000 ± 1800
*M. tuberculosis*	Maltose-1P^b^	0.25 ± 0.05	1.26 ± 0.07	5000 ± 1000
	Maltosehexaose^b^	35 ± 8	15.4 ± 1.1	440 ± 100
*S. coelicolor* Isoform I	Maltose-1P^c^	0.30 ± 0.06	12.3 ± 0.5	41000 ± 8000
	Maltohexaose^c^	1.5 ± 0.3	53 ± 2	36000 ± 7000

^a^This study.

^b^Obtained from^4^.

^c^Obtained from^15^.

**Table 2:** Data collection and refinement statistics.

Dataset	Maltose	Maltose-1-P	APO
Data collection^a^			
Beamline at DLS	I04–1	I04	I24
Wavelength (Å)	0.92	0.979	0.978
Space group	P2_1_2_1_2_1_	P2_1_2_1_2_1_	P6
Unit cell dimensions (Å)	a = 80.33; b = 113.90; c = 220.50	a = 77.5; b = 112.9; c = 221.4	a = b = 197.64; c = 105.62
Resolution range (Å)	54.3–1.92 (2.02–1.92)	221.4–3.32 (3.50–3.32)	171.2–3.13 (3.30–3.13)
Reflections (measured/unique)	147,668/10,554	149,129/28,988	563,574/41.587
Completeness (%)	99.9 (99.9)	99.8 (99.8)	99.7 (99.7)
Multiplicity	12.8 (13.7)	10.9 (8.6)	13.5 (13.6)
*R*_sym_^b^	0.065 (0.690)	0.083 (0.595)	0.218 (1.541)
Mean [(I)/σ (I)]	11.4 (2.44)	5.0 (5.2)	13.0 (2.3)
Protomers per asymmetric unit	2	2	2
Matthews coefficient (Å^3^ Da^−1^)	3.25	3.26	3.47
Solvent content (%)	62.1	62.5	67.4
Refinement			
Resolution range (Å)	37.74–1.95	100.6–3.32	57.06–3.13
*R*_factor_^c^/Free *R*_factor_^d^(%)	17.5/20.2	17.8/21.4	25.9/30.8
Unique reflections (working/test set)	10,227/564	29,449/1494	41621/2093
Water molecules	934	116	0
Total number of atoms	11650	10246	10489
r.m.s.d. bond lengths (Å)	0.010	0.010	0.013
r.m.s.d. bond angles (°)	0.990	1.210	1.832
Ramachandran plot statistics			
Residues in allowed regions (%)	97.1	93.1	90.4

^a^Values in parenthesis correspond to the outermost resolution shell.

^b^*R*_sym_ = ∑*h*∑*i* |*Ii*(*h*)-‹*I*(*h*)›|/∑*h*∑*i Ii*(*h*), where *I* is the observed intensity and ‹*I*› is the average intensity of multiple observations of symmetry-related reflections.

^c^*R*_factor_ = ∑||F_o_|-|F_c_||/∑|F_o_| where |F_o_| and |F_c_| are observed and calculated structure factor amplitudes, respectively.

^d^Free *R*_factor_ is the cross-validation *R*_factor_ computed for a randomly chosen subset of 5% of the total number of reflections, which were not used during refinement.

## References

[b1] Zumla A. *et al.* The WHO 2014 global tuberculosis report–further to go. The Lancet. Global health 3, e10–12 (2015). 25539957 10.1016/S2214-109X(14)70361-4

[b2] Decker B. K. & Palmore T. N. Hospital water and opportunities for infection prevention. Current infectious disease reports 16, 432 (2014). 25217106 10.1007/s11908-014-0432-y PMC5583638

[b3] Velayati A. A. *et al.* Emergence of new forms of totally drug-resistant tuberculosis bacilli: super extensively drug-resistant tuberculosis or totally drug-resistant strains in iran. Chest 136, 420–425 (2009). 19349380 10.1378/chest.08-2427

[b4] Kalscheuer R. *et al.* Self-poisoning of Mycobacterium tuberculosis by targeting GlgE in an alpha-glucan pathway. Nat Chem Biol 6, 376–384 (2010). 20305657 10.1038/nchembio.340 PMC3256575

[b5] Elbein A. D., Pastuszak I., Tackett A. J., Wilson T. & Pan Y. T. Last Step in the Conversion of Trehalose to Glycogen: a mycobacterial enzyme that transfers maltose from maltose 1-phosphate to glycogen. J Biol Chem 285, 9803–9812 (2010). 20118231 10.1074/jbc.M109.033944 PMC2843229

[b6] Mendes V., Maranha A., Lamosa P., da Costa M. S. & Empadinhas N. Biochemical characterization of the maltokinase from Mycobacterium bovis BCG. BMC Biochem 11, 21 (2010). 20507595 10.1186/1471-2091-11-21 PMC2885305

[b7] Chandra G., Chater K. F. & Bornemann S. Unexpected and widespread connections between bacterial glycogen and trehalose metabolism. Microbiology 157, 1565–1572 (2011). 21474533 10.1099/mic.0.044263-0

[b8] Mendes V., Maranha A., Alarico S. & Empadinhas N. Biosynthesis of mycobacterial methylglucose lipopolysaccharides. Natural product reports 29, 834–844 (2012). 22678749 10.1039/c2np20014g

[b9] Sambou T. *et al.* Capsular glucan and intracellular glycogen of Mycobacterium tuberculosis: biosynthesis and impact on the persistence in mice. Mol Microbiol 70, 762–774 (2008). 18808383 10.1111/j.1365-2958.2008.06445.x PMC2581643

[b10] Leiba J. *et al.* Mycobacterium tuberculosis maltosyltransferase GlgE, a genetically validated antituberculosis target, is negatively regulated by Ser/Thr phosphorylation. J Biol Chem 288, 16546–16556 (2013). 23609448 10.1074/jbc.M112.398503 PMC3675590

[b11] Stam M. R., Danchin E. G., Rancurel C., Coutinho P. M. & Henrissat B. Dividing the large glycoside hydrolase family 13 into subfamilies: towards improved functional annotations of alpha-amylase-related proteins. Protein engineering, design & selection: PEDS 19, 555–562 (2006). 10.1093/protein/gzl044 17085431

[b12] Ramasubbu N., Paloth V., Luo Y., Brayer G. D. & Levine M. J. Structure of human salivary alpha-amylase at 1.6 A resolution: implications for its role in the oral cavity. Acta Crystallogr D Biol Crystallogr 52, 435–446 (1996). 15299664 10.1107/S0907444995014119

[b13] Janecek S., Svensson B. & Henrissat B. Domain evolution in the alpha-amylase family. J Mol Evol 45, 322–331 (1997). 9302327 10.1007/pl00006236

[b14] Brzozowski A. M. & Davies G. J. Structure of the Aspergillus oryzae alpha-amylase complexed with the inhibitor acarbose at 2.0 A resolution. Biochemistry 36, 10837–10845 (1997). 9283074 10.1021/bi970539i

[b15] Syson K. *et al.* Structure of Streptomyces maltosyltransferase GlgE, a homologue of a genetically validated anti-tuberculosis target. J Biol Chem 286, 38298–38310 (2011). 21914799 10.1074/jbc.M111.279315 PMC3207445

[b16] Syson K. *et al.* Structural insight into how Streptomyces coelicolor maltosyl transferase GlgE binds alpha-maltose 1-phosphate and forms a maltosyl-enzyme intermediate. Biochemistry 53, 2494–2504 (2014). 24689960 10.1021/bi500183c PMC4048318

[b17] Veleti S. K., Lindenberger J. J., Ronning D. R. & Sucheck S. J. Synthesis of a C-phosphonate mimic of maltose-1-phosphate and inhibition studies on Mycobacterium tuberculosis GlgE. Bioorg Med Chem 22, 1404–1411 (2014). 24461562 10.1016/j.bmc.2013.12.058 PMC4023634

[b18] Veleti S. K., Lindenberger J. J., Thanna S., Ronning D. R. & Sucheck S. J. Synthesis of a poly-hydroxypyrolidine-based inhibitor of Mycobacterium tuberculosis GlgE. J Org Chem 79, 9444–9450 (2014). 25137149 10.1021/jo501481r PMC4201354

[b19] Lindenberger J. J., Kumar Veleti S., Wilson B. N., Sucheck S. J. & Ronning D. R. Crystal structures of Mycobacterium tuberculosis GlgE and complexes with non-covalent inhibitors. Sci Rep 5, 12830 (2015). 26245983 10.1038/srep12830 PMC4526890

[b20] Thanna S., Lindenberger J. J., Gaitonde V. V., Ronning D. R. & Sucheck S. J. Synthesis of 2-deoxy-2,2-difluoro-alpha-maltosyl fluoride and its X-ray structure in complex with Streptomyces coelicolor GlgEI-V279S. Organic & biomolecular chemistry 13, 7542–7550 (2015). 26072729 10.1039/c5ob00867k PMC4489993

[b21] MacGregor E. A., Janecek S. & Svensson B. Relationship of sequence and structure to specificity in the alpha-amylase family of enzymes. Biochim Biophys Acta 1546, 1–20 (2001). 11257505 10.1016/s0167-4838(00)00302-2

[b22] Mendes V., Maranha A., Alarico S., da Costa M. S. & Empadinhas N. Mycobacterium tuberculosis Rv2419c, the missing glucosyl-3-phosphoglycerate phosphatase for the second step in methylglucose lipopolysaccharide biosynthesis. Sci Rep 1, 177 (2011). 22355692 10.1038/srep00177 PMC3240985

[b23] Leslie A. G. W. & Powell H. R. Processing diffraction data with MOSFLM. Nato Sci Ser Ii Math 245, 41–51 (2007).

[b24] Evans P. R. & Murshudov G. N. How good are my data and what is the resolution? Acta Crystallogr D 69, 1204–1214 (2013). 23793146 10.1107/S0907444913000061 PMC3689523

[b25] Winn M. D. *et al.* Overview of the CCP4 suite and current developments. Acta Crystallogr D Biol Crystallogr 67, 235–242 (2011). 21460441 10.1107/S0907444910045749 PMC3069738

[b26] Vonrhein C. *et al.* Data processing and analysis with the autoPROC toolbox. Acta Crystallogr D Biol Crystallogr 67, 293–302 (2011). 21460447 10.1107/S0907444911007773 PMC3069744

[b27] Evans P. Scaling and assessment of data quality. Acta Crystallogr D Biol Crystallogr 62, 72–82 (2006). 16369096 10.1107/S0907444905036693

[b28] Adams P. D. *et al.* PHENIX: a comprehensive Python-based system for macromolecular structure solution. Acta Crystallogr D Biol Crystallogr 66, 213–221 (2010). 20124702 10.1107/S0907444909052925 PMC2815670

[b29] McCoy A. J. *et al.* Phaser crystallographic software. Journal of Applied Crystallography 40, 658–674 (2007). 19461840 10.1107/S0021889807021206 PMC2483472

[b30] Emsley P., Lohkamp B., Scott W. G. & Cowtan K. Features and development of Coot. Acta Crystallogr D Biol Crystallogr 66, 486–501 (2010). 20383002 10.1107/S0907444910007493 PMC2852313

