# Supplementary material for: Structure of Mycobacterium thermoresistibile GlgE defines novel conformational states that contribute to the catalytic mechanism
Source: Sci Rep. 2015 Nov 30;5:17144. doi: 10.1038/srep17144 (PMC4663749; doi:10.1038/srep17144)
Supplement: Supplementary Information [file srep17144-s1.pdf]

Supplementary information

Structure of *Mycobacterium thermoresistibile* GlgE defines novel conformational states that contribute to the catalytic mechanism.

Vitor Mendes[1,2], Michal Blaszczyk[1], Ana Maranha[2], Nuno Empadinhas[2], Tom L Blundell[1]*

1 - Department of Biochemistry, University of Cambridge, Cambridge CB2 1GA, UK

2 – Molecular Mycobacteriology Group, CNC-Center for Neuroscience and Cell Biology, University of Coimbra, 3004-517 Coimbra, Portugal.

*** - Corresponding author: tom@cryst.bioc.cam.ac.uk**

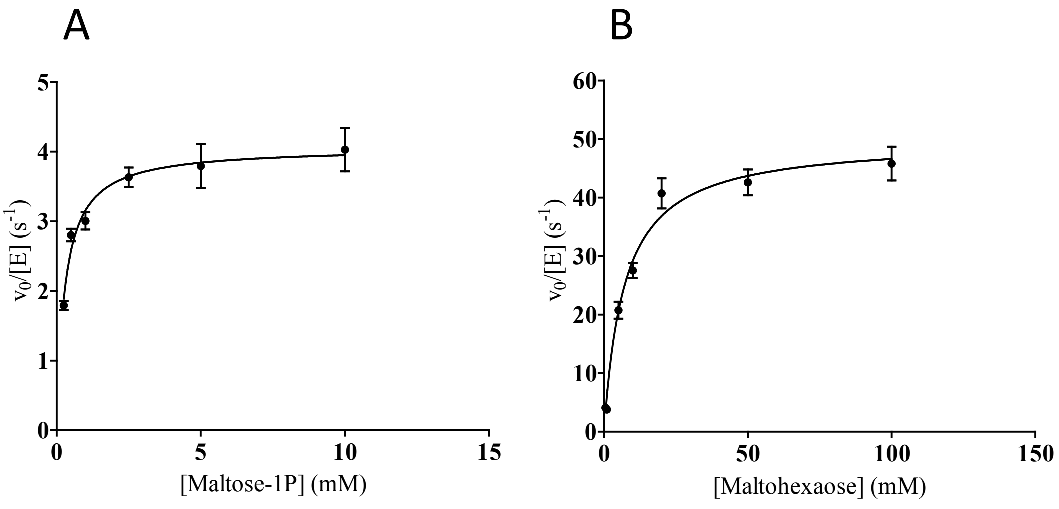

Supplementary figure S1: *M. thermoresistibile* GlgE kinetics with maltohexaose and maltose-1P. Maltohexaose concentration was kept at 1mM in A and maltose-1P concentration was kept at 5mM in B.

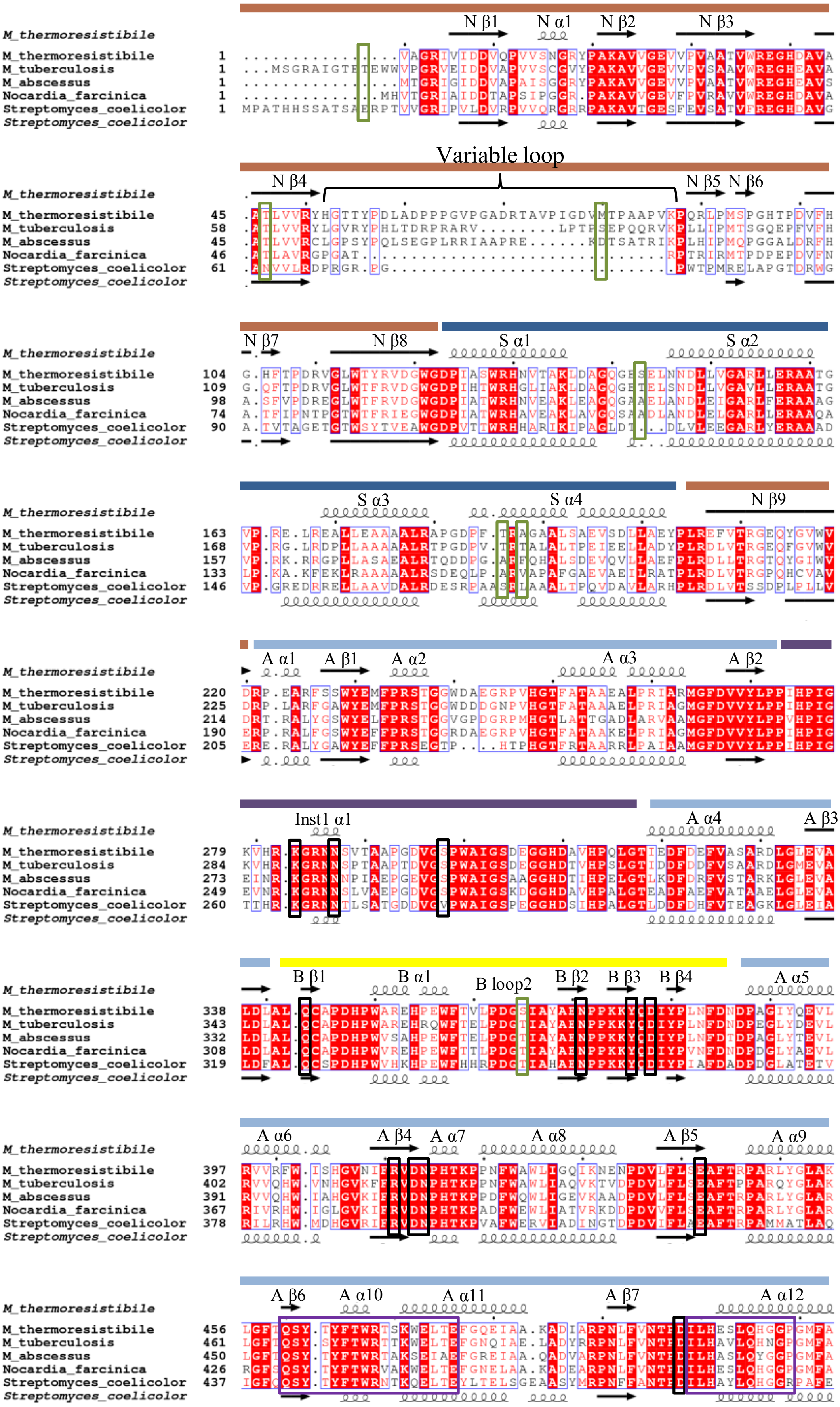

M_thermoresistibile
M_tuberculosis
M_abscessus
Nocardia_farcinica
Streptomyces_coelicolor
N β1
N α1
N β2
N β3
Variable loop
N β4
N β5
N β6
N β7
N β8
S α1
S α2
S α3
S α4
N β9
A α1
A β1
A α2
A α3
A β2
Inst1 α1
A α4
A β3
B β1
B α1
B loop2
B β2
B β3
B β4
A α5
A α6
A β4
A α7
A α8
A β5
A α9
A β6
A α10
A α11
A β7
A α12

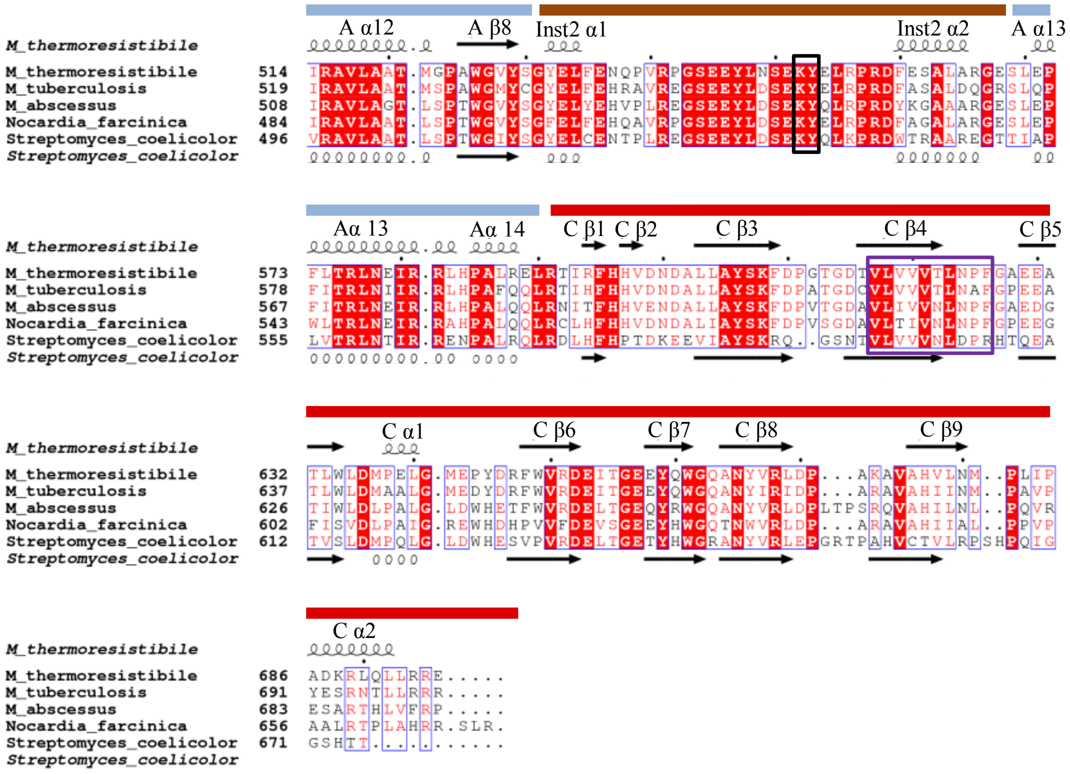

Supplementary Figure S2: Conservation of active-site and phosphorylation-site residues in GlgE orthologues. The multiple sequence alignment was performed with ClustalOmega[1,2]. Secondary structure features above the sequences correspond to *M. thermoresistiblile* GlgE maltose co-crystallization structure (5GCM). The secondary structure features indicated below correspond to *S. coelicolor* GlgE structure (3ZSS). A horizontal line in different colours represents the several domains: Domain N is represented in brown, Domain S in blue, Domain A in light blue, Domain B in yellow, Insert 1 in cyan, Insert 2 in dark orange and Domain C in red. Black boxes highlight residues that directly interact with maltose-1P. Green Boxes highlight GlgE residues know to be phosphorylated by PknB in *M. tuberculosis*[3]. Purple boxes highlight residues found to interact with linear α-glucan chains[4]. Red boxes highlight conserved residues. This figure was prepared with ESPript 3[5].

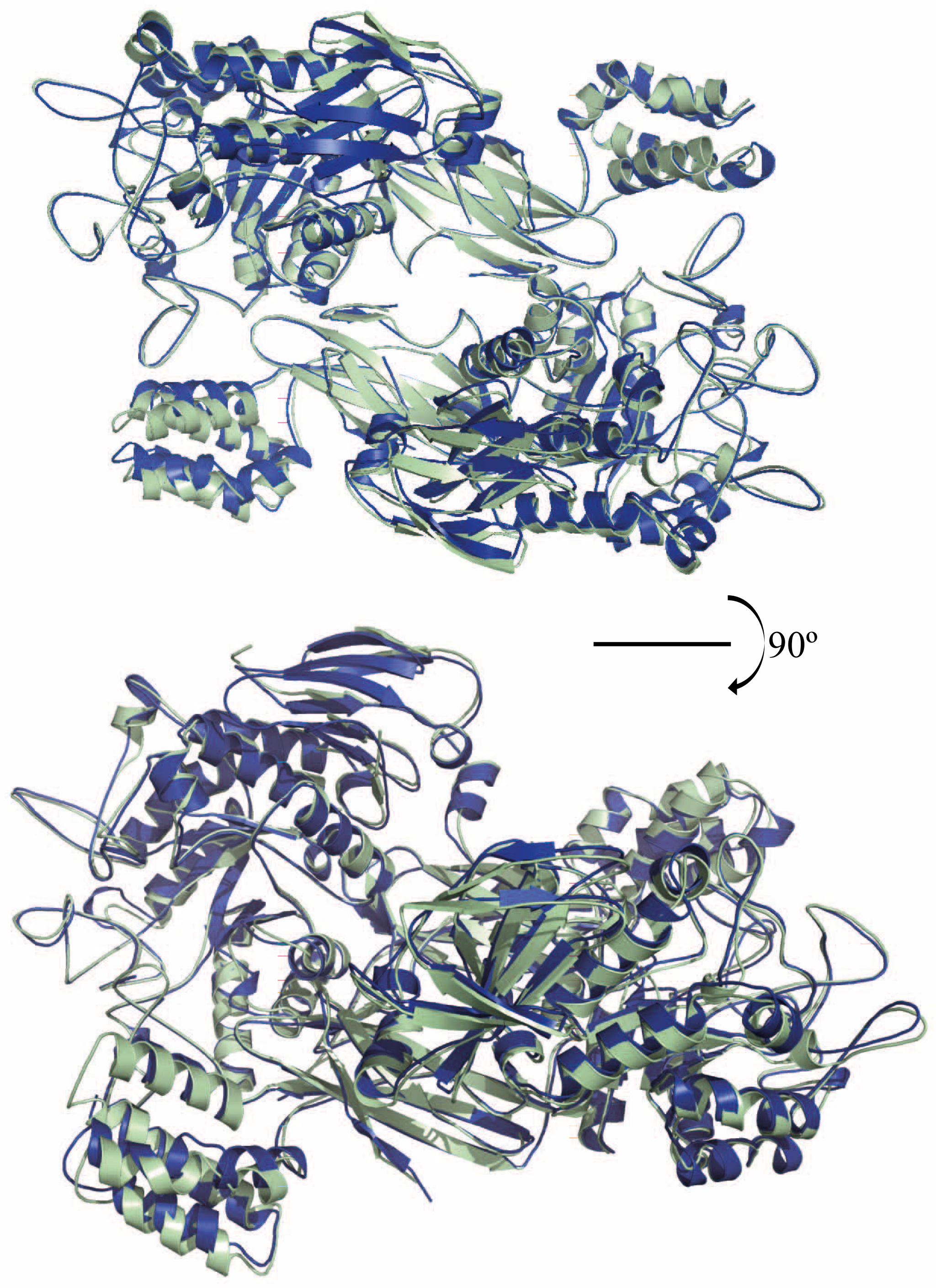

Supplementary Figure S3: Superposed structures of maltose co-crystallization (5CGM) in blue and apo form (5CJ5) in green.

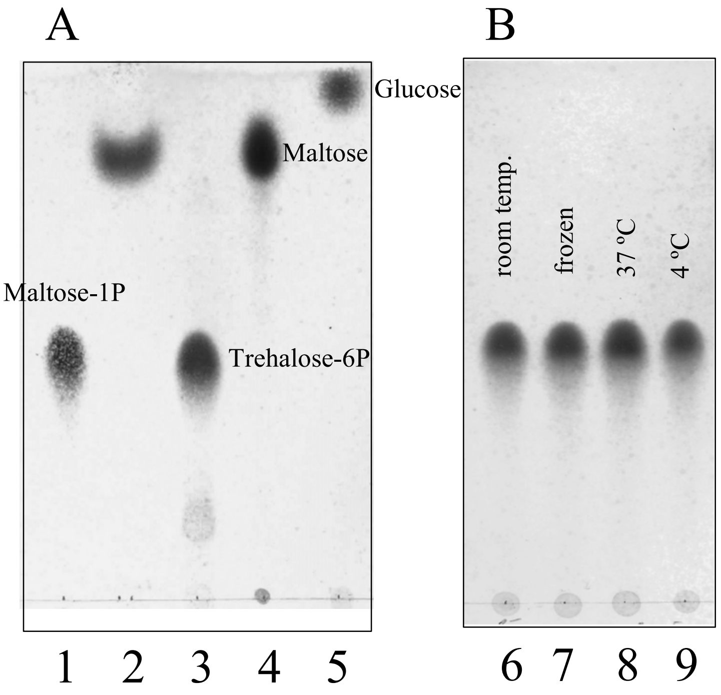

Supplementary figure S4: (A) TLC analysis of maltose, maoltose-1P, glucose and trehalose-6P. Lane 2 corresponds to maltose-1P treated with alkaline phosphatase. (B) TLC analysis of 1 week (6, 7, 9) and 1h (8) maltose-1P solutions.

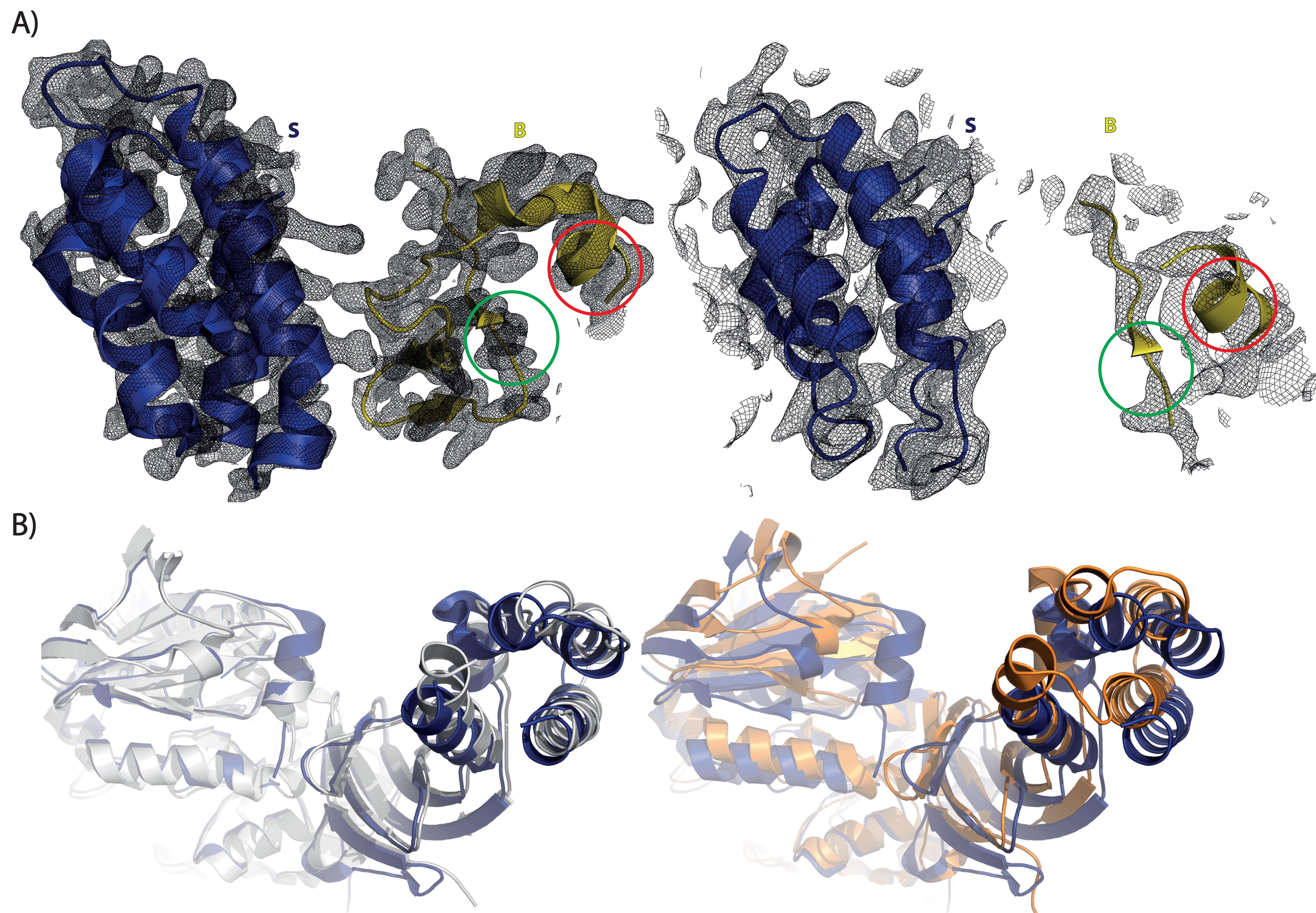
A)
S
B
S
B
B)

Supplementary figure S5: (A) Electron density maps of maltose co-crystallization structure (right panel) and maltose-1P co-crystallization structure (left panel) focusing on the S and B domains of opposing protomers. The represented S domain of the maltose-1P co-crystallization structure belongs to the maltose bound protomer. Red and green circles highlight the same regions between both panels. (B) Superposition of *M. thermoresistibile* GlgE in blue (maltose co-crystallization structure) in blue with *M. tuberculosis* GlgE (4U33) (right panel) and *S. coelicolor* GlgE (3ZT5) (left panel).

References

1 Goujon, M. *et al.* A new bioinformatics analysis tools framework at EMBL-EBI. *Nucleic Acids Res* **38**, W695-699, (2010).

2 Sievers, F. *et al.* Fast, scalable generation of high-quality protein multiple sequence alignments using Clustal Omega. *Molecular systems biology* **7**, 539, (2011).

3 Leiba, J. *et al.* Mycobacterium tuberculosis maltosyltransferase GlgE, a genetically validated antituberculosis target, is negatively regulated by Ser/Thr phosphorylation. *J Biol Chem* **288**, 16546-16556, (2013).

4 Lindenberger, J. J., Kumar Veleti, S., Wilson, B. N., Sucheck, S. J. & Ronning, D. R. Crystal structures of Mycobacterium tuberculosis GlgE and complexes with non-covalent inhibitors. *Sci Rep* **5**, 12830, (2015).

5 Robert, X. & Gouet, P. Deciphering key features in protein structures with the new ENDscript server. *Nucleic Acids Res* **42**, W320-324, (2014).
